# Unseen Impacts: Rural Adolescents’ Self-Perception and Mental Health in the Age of Dermatology-Related Social Media

**DOI:** 10.7759/cureus.92591

**Published:** 2025-09-17

**Authors:** Katheryn Bell, Elham Zayed, Ellen Ireland, Eric Reyes

**Affiliations:** 1 Department of Pediatric Dermatology, Indiana University School of Medicine, Indianapolis, USA; 2 Department of Clinical Family Medicine, Indiana University School of Medicine, Indianapolis, USA; 3 Department of Biostatistics and Health Data Science, Indiana University School of Medicine, Indianapolis, USA

**Keywords:** adolescent dermatology, adolescent mental health, influencer, medical influencer, pediatric dermatology, skincare routines, social comparison, social media, social media in medicine, tiktok skincare trends

## Abstract

Background: Adolescence represents a pivotal period in the development of self-image, with skin appearance playing a central role in self-confidence and peer acceptance. In rural communities with limited access to dermatologic care, adolescents frequently turn to social media platforms such as TikTok, Instagram, and YouTube for skin-related information. These platforms often promote idealized beauty standards and unverified treatments, potentially influencing self-perception and psychological well-being. To investigate these potential impacts, the objective of this survey-based cross-sectional study was to evaluate the psychological impact of dermatology-related social media exposure on self-image, emotional health, and professional aspirations among adolescents in a rural setting.

Materials and methods: A cross-sectional survey was administered to 206/294 high school students (response rate: 70.1%) in a rural Indiana community. The 19-item Qualtrics survey assessed demographics, social media use, exposure to dermatologic content, and self-reported psychological effects. Inclusion required current enrollment at school and at least one prior use of social media; exclusion criteria was no social media use. Responses were analyzed using descriptive statistics and chi-square tests.

Results and discussion: Of 206 respondents, most were female (74%, n = 152) and under 18 years of age (84%, n = 175). Female students were significantly more likely than males to follow skincare influencers (36.8%, n = 56 vs. 8.5%, n = 4; p = 0.001, p < 0.05). Among those who viewed dermatology-related content, 37.8% (n = 56) reported only positive effects (e.g., increased confidence), 31.1% (n = 46) reported only negative effects (e.g., heightened self-criticism), and 4.1% (n = 6) reported both positive and negative effects. Notably, while 68.9% (n = 102) acknowledged some impact on self-image, only 22.6% (n = 35) perceived effects on their mental health, suggesting a disconnect between appearance concerns and emotional awareness. Nearly half (45%, n = 74) reported greater focus on perceived flaws, yet most (59%, n = 97) denied developing an unhealthy perspective on body image. Gender- and identity-based disparities were pronounced: female and non-binary students reported higher rates of negative self-perception compared to males. Despite these risks, over half (50.6%, n = 104) identified educational benefits, and 30.6% (n = 63) noted that dermatology content inspired interest in healthcare careers. Further, just over one-third of rural respondents (n = 75, 36.4%) reported discovering a new community or interest they would not otherwise have encountered, thereby reflecting a significant integration into the lives of rural, underserved populations. These findings underscore the dual nature of aesthetic media, both reinforcing body dissatisfaction and offering pathways for learning, community, and identity exploration.

Conclusions: Dermatology-related social media exerts complex psychological effects on rural adolescents, amplifying self-image concerns while simultaneously providing educational value and career inspiration. Female and gender-diverse students appear particularly vulnerable, highlighting the need for adolescent-centered media literacy and inclusive, evidence-based dermatology messaging.

## Introduction

Adolescence is a pivotal stage for self-image development, and skin appearance often plays a central role in shaping peer relationships and self-esteem [1,2]. Social media platforms such as TikTok and Instagram amplify dermatology-related content, often presenting filtered, idealized skin images alongside influencer-endorsed treatments that may lack scientific credibility [1-3]. For adolescents in rural areas, who often have limited access to dermatologic care, these digital sources may become primary references for skincare information [4]. Prior studies have documented associations between social media and body dissatisfaction, yet little is known about the specific psychological impact of dermatologic content among rural adolescents. This study investigates that gap, examining how engagement with online skincare content influences self-perception and emotional health.

## Materials and methods

A voluntary cross-sectional survey (n = 206/294; response rate: 70.1%) was conducted in February 2025 with rural Indiana high school students ages 13-20. Eligibility was based on current enrollment status at the high school. Participants were invited to participate through classroom and school-wide intercom announcements to participate in the voluntary study, as well as emails to the students and their parents. Parents were contacted via email for student consent attainment. Data was collected through a 19-item custom Qualtrics survey assessing demographics, social media use, sources of dermatology information, and psychological impact, with answers stored on password-protected computers. Participants were informed that the survey included sensitive items (e.g., on mental health, body image, and appearance concerns) and were encouraged to stop the survey if uncomfortable with any question. No surveys were discontinued midway.

A custom survey was developed specifically for this rural targeted population, as no existing validated instruments addressed the targeted constructs. The survey was reviewed by two dermatologists, two rural-focused PhD researchers, and the school principal for clarity and appropriateness, and adjustments were made when indicated. Formal pilot testing and psychometric validation were not conducted; items were refined based on expert feedback. The lack of formal validation is acknowledged as a limitation of the study. Plans are in place to formally validate this survey in the future. Inclusion required current enrollment at the rural Indiana high school and at least one instance of self-reported prior engagement with social media; exclusion criteria were no social media use. The study was approved by the Indiana University Institutional Review Board (approval number: #25625). Parents provided written consent if students were under 18 years old, and students provided assent. Surveys required full completion before submission. Statistical analyses used descriptive statistics and chi-square tests in R version 4.4.3 (R Foundation for Statistical Computing, Vienna, Austria), with p < 0.05 considered statistically significant. Chi-square tests of independence were used when examining the relationship between distinct categorical characteristics. McNemar’s test was used to determine whether participants are equally likely to select one option over another in a “select all that apply” question.

## Results

Most participants were female (152, 74%) and under age 18 (175, 84%), as seen in Table 1. We have strong evidence (𝜒_2_^2 ^= 57.8, p < 0.001) that women engage with skin-related content on social media more often than men. We also note that overall, more students stated that they engaged with skin-related content on social media but did not follow creators (83, 40.3%) than those who stated they followed creators (61, 29.6%). One individual reported an age of 20 or greater, and one individual reported an age under 14; they were still included in the analysis, as they met the initial criteria for enrollment at this high school and at least one prior engagement with social media.

**Table 1:** Participant demographics: majority of females under 18 years of age ^1^Data are presented as n (%). Demographic characteristics of participants in the survey. Participants tended to be female and under the age of 18.

Characteristic	N = 206^1^
Age (yrs)	
Under 14	1 (0.5%)
14-15	77 (37%)
16-17	97 (47%)
18-19	30 (15%)
20 or older	1 (0.5%)
Gender	
Female	152 (74%)
Male	47 (23%)
Non-binary/third gender	5 (2.4%)
Prefer not to say	2 (1.0%)
Grade level	
Freshman	62 (30%)
Sophomore	51 (25%)
Junior	54 (26%)
Senior	38 (18%)
Other	1 (0.5%)

**Figure 1:**
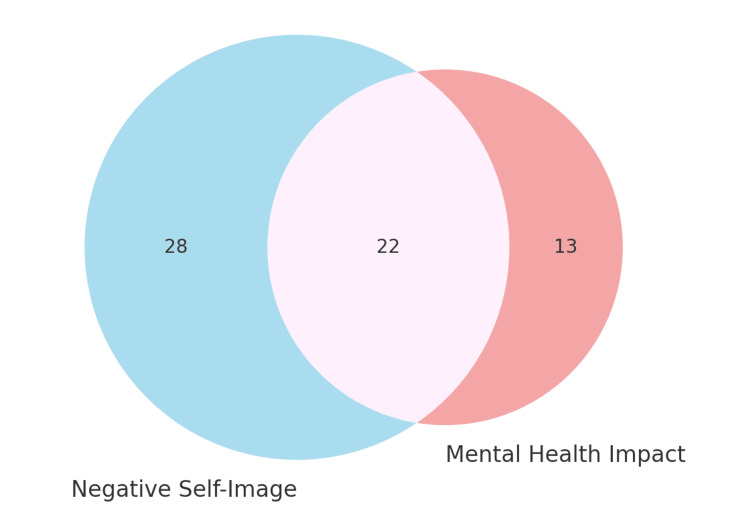
Overlap of negative self-image and mental health impact Venn diagram showing adolescents reporting negative self-image (n = 28), mental health impact (n = 13), and both (n = 22) from dermatology-related social media.

Among those who watched such content (n = 148), 37.8% (n = 56) selected only positive effects, such as increased confidence, improved self-esteem, or embracing their unique features, while 31.1% (n = 46) selected only negative impacts, such as feeling more self-critical, comparing themselves to others, or feeling less confident in their appearance, as seen in Table 2. Notably, 4.1% (n = 6) reported both positive and negative impacts. This duality shows that dermatologic content can be both empowering and distressing, often concomitantly. Mixed responses reflect the emotional complexity of aesthetic media, and overlooking them may underestimate how a single video can evoke both validation and insecurity. In contrast, of those who indicated they watched skin-related videos, only 35 (22.6%) indicated they felt the videos had any impact on their mental health, which varies quite considerably from the group that stated either positive or negative (68.9%) effects from consuming skincare content. This gap suggests that students may recognize changes in their self-image, both positive and negative, without perceiving them as changes in mental health, as shown in Figure 1.

**Table 2:** Student perceptions of the psychological impact of dermatology-related social media content Data are presented as n (%). Summary of student perceptions on the impact of dermatology videos on their psychological state.

Question	Response	Frequency
How have these skin-related videos impacted your self-esteem, body image, or overall well-being? (can choose more than one)	I do not watch skin-related videos	58 (28.2%)
	*Negatively*; they have made me feel more self-critical	36 (17.5%)
	*Negatively*; they made me compare myself to others more	36 (17.5%)
	*Negatively*; they made me feel less confident in my appearance	34 (16.5%)
	*Positively*; then boosted my self-esteem, body image, or overall well-being	33 (16%)
	*Positively*; they allowed me to embrace my unique features	29 (14.1%)
	*Positively*; they made me feel more confident in my appearance	36 (17.5%)
	*Unsure*; I am uncertain about how these videos have impacted my self-esteem or body image	44 (21.4%)
Did these videos alter your mental health?	I do not watch skin-related videos	54 (26.2%)
	*No*; they did not alter my mental health	122 (59.2%)
	*Yes*; they made me feel more anxious	28 (13.6%)
	*Yes*; they made me feel more depressed	14 (6.8%)
Have these videos led you to focus more on your perceived flaws?	I do not watch skin-related videos	43 (20.9%)
	No	59 (28.6%)
	Unsure	30 (14.6%)
	Yes	74 (35.9%)
Do you feel like you've developed an unhealthy perspective on your body or appearance after watching these videos?	I do not watch skin-related videos	42 (20.4%)
	No	97 (47.1%)
	Unsure	24 (11.7%)
	Yes	43 (20.9%)

## Discussion

The majority of respondents, 45% (n = 74), reported that skin-related videos made them focus more on their perceived flaws, while 18% (n = 30) were unsure. In contrast, most students, 59% (n = 97), indicated that these videos did not give them an unhealthy perspective on their body or appearance, suggesting that many do not equate an increased focus on perceived flaws with developing a negative body image.

This dissociation may reflect developmental limitations in recognizing internal distress or stigma-related barriers, particularly in rural settings, to reporting emotional struggles [5,6]. These findings align with previous research showing adolescents exposed to appearance-focused media often experience reduced body satisfaction without labeling these feelings as anxiety or depression [3]. With most respondents being female (n = 152, 73.8%), the gender gap in psychological response is particularly striking. Female students were significantly more likely than males to report negative self-image effects (χ_2_² = 7.01, p = 0.0081, p < 0.05). These findings align with prior studies suggesting that adolescent females are more attuned to appearance-based media and are more likely to seek skincare advice online [7-11].

Additionally, non-binary students demonstrated elevated vulnerability, with three of five reporting negative self-image effects from skin-related content online. This pattern suggests adolescent girls and gender-diverse youth may be especially vulnerable to appearance-based comparison and emotional distress, particularly in rural areas where stigma and access to mental health and dermatology care remain barriers [5,7,8]. These findings highlight the need for nuanced language and early screening tools when evaluating media-related psychological outcomes, as self-image concerns may signal emerging mental health risk [12,13].

Importantly, the impact of dermatologic content was not universally negative. Over half (n = 104, 50.6%) acknowledged that such content offered both educational value and potential harm. Additionally, 30.6% (n = 63) reported that viewing dermatology-related social media sparked interest in healthcare careers, including dermatology, medicine, and skincare. Similar studies have noted that social media can serve as a supportive space for exploring identity and connecting with others who share similar dermatologic challenges [13-15]. Similarly, just over one-third of rural respondents (n = 75, 36.4%) reported discovering a new community or interest they would not otherwise have encountered-reflecting a particularly meaningful integration into the lives of rural, underserved populations. Our findings highlight the complex role of digital media: while it can contribute to body dissatisfaction and appearance-related anxiety, it also provides opportunities for education, inspiration, connection, and career exploration.

Our findings show that concern for misinformation on social media remains high in rural teens, with 58.7% (n = 120) expressing worry about the spread of misinformation; however, 23.3% (n = 48) believe it causes more good than harm. Although concerns about misinformation remain pervasive across unregulated platforms, social media has also been recognized for its potential to strengthen the physician-patient relationship [16-18]. In line with our findings that exposure to dermatology content may inspire interest in healthcare careers, online engagement can also encourage self-advocacy by prompting patients to bring new questions and ideas to clinical encounters [16-18]. A substantial portion (n = 84, 40.8%) of rural adolescents report discussing skincare products and influencers with their peers, underscoring the importance of grounding these conversations in accurate, evidence-based information to counter misinformation and support healthier choices. Social media also serves as a frequent source of information about diagnoses, symptom triggers, and treatment options before or after clinical encounters, which can foster more informed discussions, strengthen patient engagement, and ultimately enhance the quality of clinical interactions [4,12,13,16].

This study has several limitations. The sample was drawn from a single rural high school, which limits generalizability to other settings. Additionally, the cohort’s demographics, predominantly female and under 18, further constrain representativeness. The lack of formal validation of the survey is a general limitation; however, the survey was reviewed by two dermatologists, two rural-focused PhD researchers, and the school principal for clarity and procured revisions. Dermatologic diagnoses were collected, but baseline mental health and socioeconomic status were not measured, representing potential confounders. However, the dermatologic diagnoses were not statistically controlled for in multivariate models, which may limit the ability to fully isolate the independent effects of social media exposure on self-perception. Future studies with larger sample sizes could incorporate regression-based approaches to adjust for these variables. No adjustments for multiple comparisons were made, as only a small number of chi-square analyses were conducted, and potentially confounding variables such as socioeconomic status and baseline mental health were not collected.

Additionally, data were self-reported, introducing recall and social desirability bias, and some students were inconsistent when reporting exposure to skin-related content, which may cause classification error. Because participants were from the same school community, peer influence and shared networks may have shaped their responses, potentially reducing the independence of the observations. Lastly, the cross-sectional design precludes causal inference between exposure to dermatology-related social media and psychological outcomes. Future studies with multi-site recruitment, longitudinal follow-up, and objective measures are needed to enhance validity.

## Conclusions

Dermatology-related social media content has a nuanced influence on rural adolescents. Many reported heightened focus on self-image without recognizing broader effects on mental health, underscoring a disconnect between appearance concerns and emotional awareness. Female and gender-diverse students appeared especially vulnerable, highlighting the need for adolescent-centered media literacy efforts. At the same time, students identified educational value and even career inspiration through these platforms. By engaging as content creators, health professionals can help provide inclusive, evidence-based messaging that supports healthy self-image, realistic expectations, and meaningful patient engagement during a formative stage of development.

## Appendices

**Table 3:** Survey instrument titled "Survey: Dermatology and Social Media," consisting of 29 questions across five sections. Patients were required to complete all questions before submission MD: doctor of medicine, DO: doctor of osteopathic medicine, PA: physician assistant, NP: nurse practitioner, RN: registered nurse, MA: medical assistant

Survey: dermatology and social media	
Purpose: This is a survey about how skin care videos on social media make you feel and affect your choices, including online activity and mental health.	
Instructions: Please answer the following questions as honestly as possible. Your responses will remain confidential.	
Section 1: demographic information	
1. Age	
	Under 14
	14-15
	16-17
	18-19
	20 or older
2. Gender	
	Male
	Female
	Non-binary/third gender
	Prefer not to say
3. Grade level	
	Freshman
	Sophomore
	Junior
	Senior
Section 2: knowledge and perceptions	
4. Do you follow/receive content regarding skincare/good dermatology practice on social media?	
	Yes, I receive skin-related content and follow skin-related creators
	Yes, I receive skin-related content, but I do not follow skin-related creators
	No, I do not receive skin-related content and do not follow skin-related creators
5. Do you get skincare recommendations on social media from (check all that apply):	
	Licensed MD/DO (medical doctors)
	Advanced practice providers (PA, NP)
	Medical students/PA students
	Estheticians (RNs, MAs)
	Makeup artists
	General “influencers”
	Friends making videos online
6. Who do you prefer to receive skincare recommendations from? (check all that apply)	
	Videos online
	Websites/online search
	From a doctor/licensed professional in person
	From friends
	I don’t know, haven’t considered
7. What proportion of your online videos are linked with sponsorships?	
	Under half
	Over half
	Unsure
	Probably more than I realize
8. When you watch a video, do you look to see the creator’s credentials?	
	Yes
	Sometimes
	No
	I do not watch skin-related videos
9. When you watch a video, do you care if the creator is not a licensed healthcare professional?	
	Yes, I only listen if they are a verified healthcare professional
	Yes, but I will listen if they cite verified sources for their answer
	Yes, but if they say “they heard it from a dermatologist/friend/colleague,” it does not matter to me if they themselves are not verified healthcare providers
	No, I am indifferent
	No, but I believe I should
	I do not watch these videos
Section 3: behavior and habit changes	
10. Have you initiated a skincare routine after watching a video about skincare?	
	Yes
	No
11. If yes, what changes did you make? (check all that apply)	
	Added whole “skincare routines”
	Added a moisturizer
	Added a vitamin C serum
	Added retinol
	Added facial scrubs
	Created home-based remedies
	Other: ______
12. What have you changed about your lifestyle after interacting with social media dermatologic content? (check all that apply)	
	Water intake
	Diet (e.g., avoiding processed sugar, dairy, inflammatory foods)
	Exercise routine
	Added vitamins or supplements
	I have not seen any dermatology-related video/social media post
13. Did seeing videos online prompt you to seek further medical help regarding skincare (e.g., making an appointment)? (check all that apply)	
	Yes, I needed more guidance
	Yes, I did because a product caused skin damage
	No
	I have not seen any dermatology-related video/social media post
Section 4: psychological impacts	
14. Do you experience any of the following? (check all that apply)	
	Acne
	Blackheads
	Redness
	Dry skin
	Oily skin
	Eczema
	Other: _____
15. How have these skin-related videos impacted your self-esteem, body image, or overall well-being? (check all that apply)	
	Positively, they boosted my self-esteem, body image, or overall well-being
	Positively, they made me feel more confident in my appearance
	Positively, they allowed me to embrace my unique features
	Negatively, they have made me feel more self-critical
	Negatively, they made me feel less confident in my appearance
	Negatively, they made me compare myself to others more
	Unsure, I am uncertain about how these videos have impacted my self-esteem or body image
	I do not watch skin-related videos
16. Did these videos alter your mental health? (check all that apply)	
	Yes, they made me feel more anxious
	Yes, they made me feel more depressed
	No, they did not alter my mental health
	I do not watch skin-related videos
17. Have you found yourself checking social media videos more frequently?	
	Yes
	No
	Unsure
	I do not watch skin-related videos
18. Have these videos made you wish to use photo-editing apps to alter your appearance?	
	Yes
	No
	Unsure
	I do not watch skin-related videos
19. Have you felt distressed about the financial impact of maintaining an extensive skincare routine?	
	Yes
	No
	Unsure
20. Have these videos led you to focus more on your perceived flaws?	
	Yes
	No
	Unsure
	I do not watch skin-related videos
21. Do you feel like you’ve developed an unhealthy perspective on your body or appearance after watching these videos?	
	Yes
	Unsure
	No
	I do not watch skin-related videos
Section 5: psychosocial impacts	
22. Have you found a community or newfound interest when engaging with social media content that you would not have otherwise been exposed to?	
	Yes
	No
23. Have these videos made you interested in a career in dermatology, medicine, or skincare that you would not have otherwise considered?	
	Yes
	No
	I do not watch skin-related videos
24. Is skincare a part of your social circle?	
	Yes
	No
25. Do you talk with your friends about skincare products or skincare influencers that you see on social media?	
	Yes
	No
	I do not watch skin-related videos
26. If so, since discovering skincare videos online, has the topic of “skin care” taken up more of your time and mindspace?	
	Yes
	No
	Unsure
	I do not watch skin-related videos
27. Do you generally like the recent uptick in dermatology content on social media for its educational benefit, or do you think it is contributing to body-image issues across our nation? (check all that apply)	
	I like it for its educational benefit
	I think it is contributing to body-image issues
	I think it is creating a more anxious/depressed world
	Both
	Neither, I do not like these videos
	Neither, I do not watch these videos
28. Do you worry the posts you observe online are spreading misinformation?	
	Yes
	No
	Never thought about it
Thank you for participating in this survey! Your responses are valuable in helping us understand the impact of social media on skincare practices and mental health.	
